# Alkaline peptone water enrichment with a dipstick test to quickly detect and monitor cholera outbreaks

**DOI:** 10.1186/s12879-017-2824-8

**Published:** 2017-11-21

**Authors:** Godfrey Bwire, Christopher Garimoi Orach, Dauda Abdallah, Amanda Kay Debes, Atek Kagirita, Malathi Ram, David A. Sack

**Affiliations:** 1 Department of Community Health, Ministry of Health, Kampala, Kampala, Uganda; 2 Department of Community and Behavioral Sciences, Makerere University School of Public Health, College of Health Sciences, Kampala, Uganda; 3 Kasese District Local Government, Kampala, Uganda; 4 Department of International Health, DOVE Project, Johns Hopkins Bloomberg School of Public Health, Baltimore, MD USA; 5 Uganda National Health Laboratory Services (UNHS/CPHL), Ministry of Health, Kampala, Uganda

**Keywords:** *Vibrio cholerae*, Rapid test, Dipstick, Alkaline peptone water, RDT, Outbreak detection, Epidemic monitoring

## Abstract

**Background:**

Detection, confirmation and monitoring of cholera outbreaks in many developing countries including Uganda is a big challenge due to lack of the required resources and the time the test takes. Culture method which takes 24–48 h to get the feedback and requires highly skilled laboratory staff plus other complex resources is the standard test.

This study evaluated the new cholera rapid detection method that relies on *Crystal VC* dipsticks after enrichment with alkaline peptone water (APW) against the culture method for monitoring the progress of cholera outbreaks in rural setting.

**Methods:**

We conducted the study between March and June 2015. Fresh stool samples and rectal swabs were incubated in 1% APW for 6 h at room temperature before testing with RDT following the manufacturer’s instruction. The same stool sample was cultured to isolate *V. cholerae* in the standard manner. We also reviewed patient registers to epidemiologically describe the cholera epidemic.

**Results:**

We tested stool from 102 consenting suspected cholera patients reporting during daytime at Bwera Hospital (*n* = 69), Kilembe Mines Hospital (*n* = 4) and Kinyabwama Health Centre (*n* = 29). Ninety one (91) samples were positive and nine samples were negative according to both methods. One (1) sample was positive only by dipstick and one sample was positive only by culture (sensitivity of 99%, specificity of 90%, Positive Predictive Value of 99% and Negative Predictive Value of 90%). Overall, 146 suspected cholera cases and two deaths, (case fatality rate of 1.36%) were recorded during the study period. Among the cases aged 1–9 years, 63% (50/79) were males while in those aged 20–49 years, 76% (34/45) were females.

**Conclusions:**

Our findings showed that the modified dipstick test after enrichment with 1% APW had high level of accuracy in detection of *V. cholerae* and is quick, affordable alternative cholera outbreak monitoring tool in resource constrained settings. However, culture method should remain for cholera epidemic confirmation, for monitoring of antibiotic sensitivity and for production of pure isolates for molecular characterization. Further studies should be done to better understand the observed age and sex case distribution, in Kasese district.

## Background

During the past four decades Uganda has experienced several cholera outbreaks in various regions of the country. Though cholera is a potentially preventable diarrheal disease, it continues to be a major public health problem in many rural districts in Uganda [1–6].

Cholera detection, confirmation and monitoring in Uganda and many other developing countries is by laboratory isolation of *V. cholerae* bacteria by culture method [7]. The culture method has several challenges namely; it takes 24–48 h to get the test results, requires highly skilled laboratory staff who may not be available in most rural areas in Africa [8], many supplies and good laboratory infrastructure particularly electricity [9, 10] to operate the equipments (incubators). There are only a few health facilities with the capacity to confirm cholera by culture in Africa; thus, samples are sent to a central laboratory whenever possible, but this takes additional time, sometimes weeks to obtain results [11]. If available and reliable, rapid diagnostic tests (RDTs) can facilitate the reporting, detection and monitoring of the progress of cholera outbreaks since they do not require complex infrastructure or highly skilled personnel, though some training is needed [12]. The RDTs also reduce the need for unnecessary laboratory tests and facilitate rapid implementation of appropriate infection control measures which is key in preventing infection spread [13].

The introduction of RDTs in malaria treatment in Uganda prevented misdiagnosis, improved patient care and saved government funds in medicine wastage associated with syndromic treatment of cases [14]. There is no similar reliable test for cholera and diagnosis is largely syndromic which is open to inclusion of patients with similar symptoms like cholera.

There are numerous challenges in detection and monitoring of cholera outbreaks that include long distances from the central laboratory (this laboratory is usually located in the capital city) to the cholera affected communities, inadequate laboratory supplies and lack of cholera specimen transport media among others. In addition, the normal practice after confirmation of the first 10–20 stool samples is to depend on syndromic diagnosis as recommended by World Health Organization [15]. This syndromic diagnosis is open to over or underestimation of cases in countries with high incidence of diarrhea such as Uganda where acute watery diarrhea is ranked 5^th^ major cause of morbidity and mortality [16].

Unfortunately, unlike for malaria and human immuno-deficiency virus (HIV) infections, the currently available cholera rapid diagnostic testing methods are open to false positives which affect their reliability [17, 18]. Therefore, there is a need to improve and evaluate the accuracy of cholera RDTs to alert health workers in low resource countries to institute measures to prevent and control cholera outbreaks in timely manner.

This study evaluated a modification of the *Crystal VC* RDT test procedure after enrichment of stool sample in 1% alkaline peptone water (APW) against the culture method, the standard cholera confirmation and outbreak monitoring method in Uganda. Furthermore, the high *V. cholerae* detection level among the collected stool samples allowed for accurate epidemiological description of the outbreak in terms of the affected persons, place of origin, and the time of onset which provided a better understanding of factors responsible for the propagation of the epidemic in the population. This in return contributed to better cholera prevention and control efforts in the district.

## Methods

### Study design

We carried out a comparative study during a cholera outbreak that occurred between 14^th^ March and 26^th^ June 2015 in Kasese district, Western Uganda. We used a modified dipstick cholera RDT, *Crystal VC,* and compared it with the standard culture method for cholera detection, confirmation and monitoring which was available in this district. *Crystal VC* is a trade name for the cholera testing dipsticks produced by *Span Diagnostics Limited,* Surat, India.

In addition, we reviewed information in the patient registers to epidemiologically describe the epidemic so as to guide cholera prevention and control activities in the district.

### Study area

The study was conducted in Kasese district. According to Uganda Bureau of Statistics (UBOS), Kasese district had projected population derived from 2014 census of 702,029 persons [19]. The district is located in the remote part of Uganda. Kasese district shares a border with Eastern Democratic Republic of Congo (DRC), a border region known to have recurrent cholera outbreaks [20]. Suspected cholera cases seen at the three health facilities of Bwera Hospital, Kilembe Mines Hospital and Kinyabwamba Health centre III were enrolled in the study. Health facilities in Kasese district were purposively selected because previous studies indicated that Kasese was a district at high risk for cholera, with repeated cholera outbreaks [1, 21]. Furthermore, Kasese district had hospitals with the capacity to accurately carry out both the dipstick test and culture fecal specimens for cholera.

### Study population and inclusion criteria

We only included admitted consenting suspected cholera patients seen in the three health facilities. For the patients to be admitted in the treatment facilities, the health workers used the national standard case definition for cholera that was adopted from WHO guideline for cholera [22]. In this guideline a suspected cholera case is defined as “in an area where the disease is not known to be present, a patient aged 5 years or more who develops severe dehydration or dies from acute watery diarrhea,” or “in an area where there is a cholera epidemic (based on laboratory isolation of *V. cholerae* organisms and official declaration by the Ministry of Health, Kampala), the occurrence in a patient aged 2 years or more of acute watery diarrhea, with or without vomiting.”

We excluded suspected cholera cases seen at the health facilities without capacity to carry out cholera testing on all reported cholera cases including culture confirmation and also excluded cases included in the register, but not meeting the standard case definition as per national cholera prevention and control guidelines.

### Stool collection procedures

Stool samples were collected from patients following the national standard protocol for cholera stool sample collection [23]. Fresh stool samples or rectal swabs were collected from the patients by the health workers in the cholera treatment centre on arrival and before administration of antibiotic treatment and put in stool containers where they were immediately taken to the laboratory for testing and or culture. Laboratory testing was performed by trained personnel following the study protocol.

### Procedure for the enriched RDT and culture

To conduct the RDT, the fresh stool sample (2 drops) or rectal swab was incubated in 1% APW for 6 h at room temperature (20–30 °C with an average of 25 °C) from which four drops of APW were drawn and put into a test-tube. Test was conducted following the manufacturer’s instruction. To test for cholera, one *Crystal VC* RDT test strip was inserted into the test tube with 1% APW in vertical position and the test read after 10–15 min but not beyond 15 min. The test reading was interpreted as negative, positive for O1, positive for O1 and O139, positive for O139 or invalid. The results were recorded immediately in the laboratory log book and on the data collection form which was sent back to the cholera treatment centre to serve as preliminary laboratory report. Patients found to be negative with the rapid test received the same recommended medical care as those who were positive for the test.

While incubating the stool samples in preparation for testing with the RDT the other portion of the stool sample was processed for cholera culture as outlined in the standard operation procedure for cholera identification [23]. After 24–48 h, the laboratory personnel read and recorded the culture results in the laboratory register and on the study forms. The culture results were also sent to cholera treatment centres as the final laboratory report.

### Quality control and further testing at Central Public Health Laboratory (CPHL)

To ensure quality of the test results, every 10^th^ of positive sample had its isolate inoculated into a labeled Cary Blair transport media covered and packed into ice box before sending them to the Central Public Health Laboratory in Kampala to confirm the results for the purpose of quality control. Likewise, every 10^th^ of the negative samples were shipped to CPHL for quality testing and confirmation. All isolates were also sent to CPHL for further testing (serotyping) to ascertain the causative agent and serotyping. Serotyping was conducted using both polyvalent and monovalent antisera on fresh isolates following the national standard laboratory guidelines [23].

### Study variables, data storage and analysis

Data was collected on age, sex, place of origin of the cholera cases, test results for enriched RDT test (positive, negative or invalid) and the culture results. The data was coded and stored on the spreadsheet. The data was analyzed to determine percentages, sensitivity, specificity, case fatality rates (CFR) and attack rates (AR). To calculate the attack rates we used population projection estimates for the sub-counties that were derived from 2014 national population and housing census conducted by Uganda Bureau of Statistics [19]. Data analysis was by use of spreadsheet and graphpad http://www.graphpad.com. The analysis was presented in tables and graphs. Spatial distribution of the cases and deaths was done using the shapefiles for administrative boundaries from Humanitarian Data Exchange website: https://data.humdata.org. The map showing the spatial distribution of cholera cases was produced using Arc GIS software; https://www.arcgis.com.

## Results

### Laboratory results and interpretation

Overall, 70% (102/146) of suspected cholera cases seen during the study period, 14^th^ March to 26^th^ June 2015 had their stool tested using a modified RDT and the culture method. The laboratory results were as shown on Table 1.

**Table 1:** Interpretation of 1% APW enriched test and culture results for stool samples tested during the study period, 14^th^ March to 26^th^ June 2015

	Standard Diagnostic test (culture)		
Enriched test with 1% APW	Positive	Negative	Total
Positive	91	1	92
Negative	1	9	10
Total	92	10	102
Sensitivity	98.9%, (95% CI: 94.09–99.97%)		
Specificity	90%, (95% CI: 55.5–99.75%)		
Positive Predictive Value (PPV)	98.9%		
Negative Predictive Value(NPV)	90.0%		

Of the 92 samples positive by RDT, one was also positive for *V. cholerae* O139. This sample was sent to CPHL, Kampala and found to be positive *for V. cholerae* O1 *Inaba*. There were no *V. cholerae O139* detected by culture. Further laboratory tests by serotypying of the *V. cholerae* isolates sent to CPHL, Kampala from Bwera Hospital laboratory showed that they were all *V. cholerae* O1 *Inaba* serotype.

### Quality control and serotyping results

All ten (10) positive isolates sent for quality control testing in Kampala turned out positive for *V. cholerae* and the two negative specimens were negative for *V. cholera*e. Further serotyping with polyvalent (*poly Inaba Ogawa*) and *monovalent Inaba* showed positive for all isolates.

### Epidemiological description of the cholera outbreak

The cholera outbreak in Kasese district started on 14^th^ March 2015 and the last suspected cholera case was admitted on 26^th^ June 2015. A total 146 cases and two deaths, (case fatality rate of 1.36%) were reported during the study period in the three treatments centre of Bwera Hospital *n* = 107, Kilembe Hospital *n* = 4 and Kinyabwamba Health centre III *n* = 35.

**Fig. 1:**
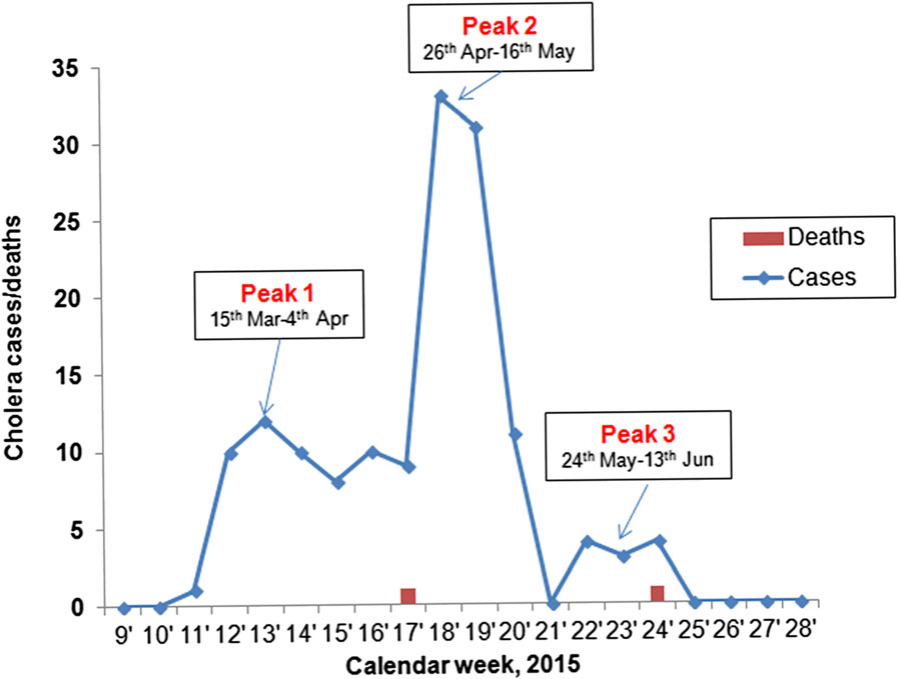
Weekly reported cholera cases and deaths in the three cholera treatment centres in Kasese district, March–June 2015

This cholera outbreak lasted for 16 weeks, had three peaks with the highest peak occurring during the 19^th^ calendar week (26^th^ April–9^th^ May 2015). The weekly number of reported cholera cases and deaths were as shown in Fig. 1.

During this outbreak there were two deaths. The first death occurred on 20^th^ April 2015 of a patient who was brought late at Bwera Hospital CTC and on examination was unconscious with severe dehydration and died few hours after admission despite intensive care from the medical team. The second death was a patient who was misdiagnosed and admitted in Bwera Hospital medical ward at night without undergoing full screening to exclude cholera. Given the inadequate monitoring at night on the medical ward, the patient deteriorated and died. Rectal swab and culture was done on the body after the death. Both the enriched RDT and culture were positive for *V. cholerae.* In response to these findings, Bwera Hospital management conducted an audit and took corrective action to prevent similar future scenario.

**Fig. 2:**
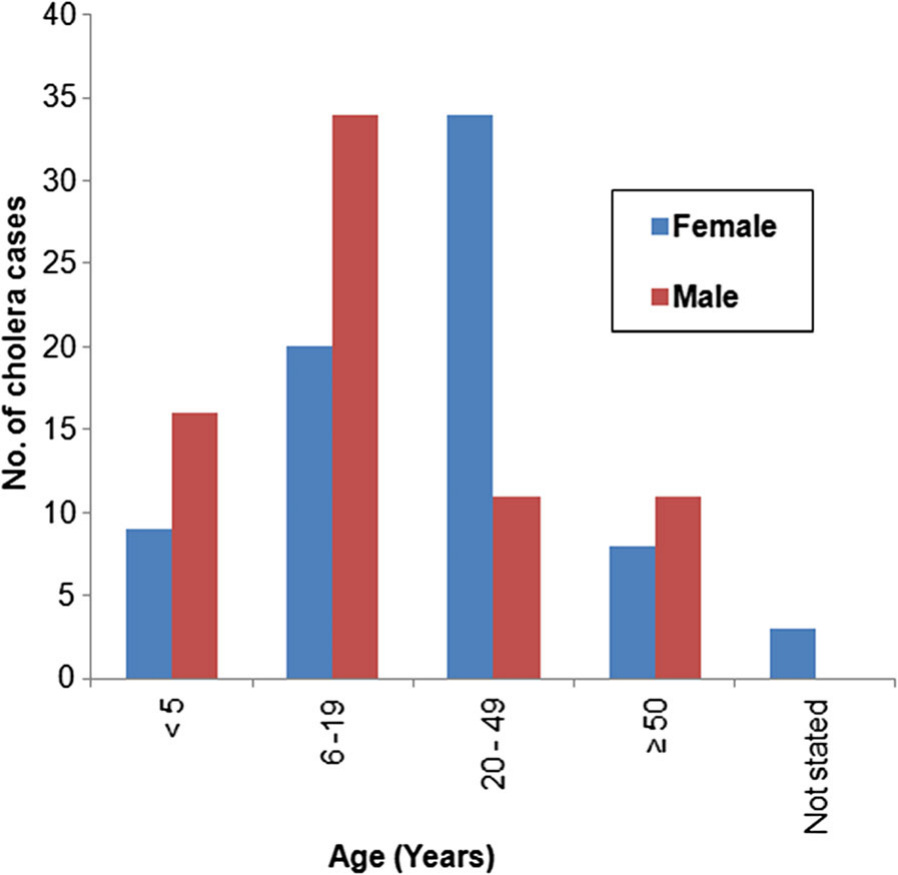
Age and sex of the suspected cholera cases recorded in the three cholera treatment centres in Kasese district, March–June 2015

In this outbreak, all age groups and both sexes were affected. In the younger age group (1–19 years) there were more males 63% (50/79), but among young adults (20–49 years) there were more females 76% (34/45). The age and sex of the cases were as shown in Fig. 2.

### Distribution of cholera cases by place of origin

The 2015 projected population of Kasese, was 702,029 persons, but the cases came from 33% (10/30) sub-counties in the district. The affected sub-counties had a total projected population of 263,064 persons. The affected sub-counties with respective number of cases and cholera attack rates are shown in Table 2.

**Table 2:** Cholera attack and case fatality rates stratified by the sub-counties of origin of cases during cholera outbreak in Kasese district, March–June 2015

Place or Subcounty of Origin	Number of cases	Number of Deaths	Projected Population	Attack Rate Per 10,000	Case Fatality Rate
DRC	5	0	–	0.0	0.0%
Ihandiro	20	0	13,549	14.8	0.0%
Isango	3	0	8099	3.7	0.0%
Karambi	19	1	8099	23.5	5.3%
Katwe TC	1	0	6411	1.6	0.0%
Bwera	7	0	17,455	4.0	0.0%
MLTC	28	1	51,018	5.5	3.6%
Mukunyu	1	0	1854	0.3	0.0%
Maliba	8	0	47,585	1.7	0.0%
Kitswamba	31	0	30,009	10.3	0.0%
Nyakiyumbu	13	0	31,854	4.1	0.0%
Kitholhu	10	0	17,131	5.8	0.0%
Total	141	2	263,064	5.4	1.4%

Note that five (5) patients came from the neighboring country, Democratic Republic of Congo (DRC)

**Fig. 3:**
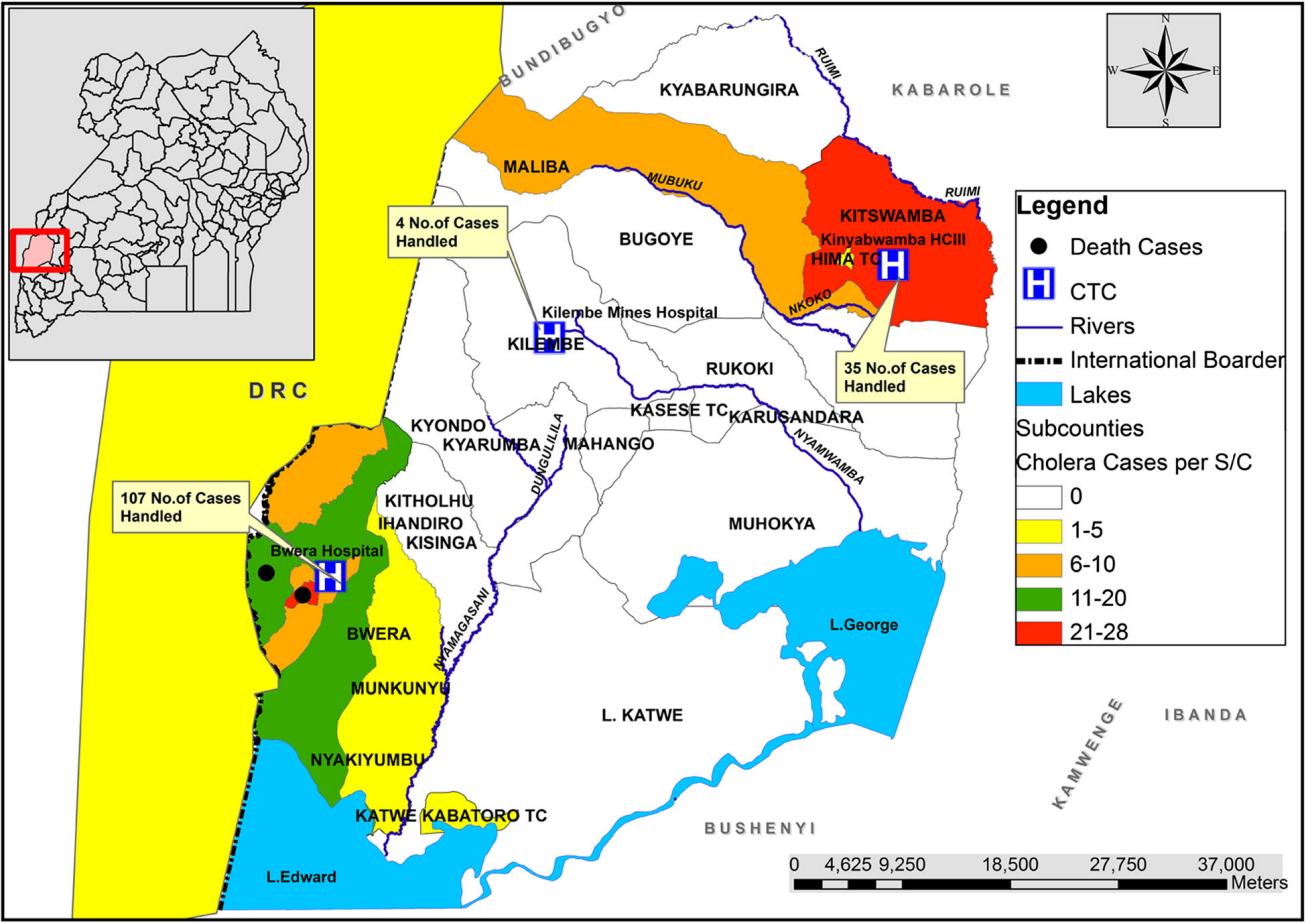
The map of Kasese district showing distribution of cholera cases by their places of origin that were treated at the three cholera treatment centers, March–June 2015

Most of the cases came from the nearby sub-counties, close to the hospitals as shown in Fig. 3

## Discussion

Our study showed that the enriched dipstick test was able to rapidly detect cholera patients and simplify the monitoring of the outbreak progress in this remote part of Uganda. By using the enrichment method, the high sensitivity, specificity, positive and negative predictive values suggest that this test procedure provides reliable results for both outbreak detection and monitoring.

Furthermore, the test is affordable (each dipstick costing USD 1.9 compared to USD 4 for culture) [18], takes shorter time and can be done in rural typical African setting saving the health workers the costly sample transportation procedures.

This enriched dipstick test is a good alternative to the expensive culture method for monitoring of cholera outbreaks and is game changer for resource constrained countries like Uganda [24] with limited laboratory infrastructure, personnel and transportation challenges.

The significance of this innovation lies in the fact that, the key reagent (alkaline peptone water) required for this rapid test is part of the normal laboratory supplies and will not require special order and importation. Also *Crystal VC* dipsticks are not new, they have been around for over two decades [17] and can easily be procured and stored. What is new is the technique of using the two components to detect and monitor cholera outbreaks more accurately and at lower or affordable cost by communities with inadequate funds and laboratory infrastructure.

It should be noted that *Crystal VC* dipsticks when used directly with stool, false positives have been reported commonly [17, 18] but when fecal specimens are first incubated in 1% APW, false positive results were found to be rare. The enriched dipstick method is not a bedside test, but results are generally available the same day as compared to 24–48 h required for culture method. Most importantly, the test is simple and can be performed even in remote areas without a microbiology laboratory or highly skilled technicians. These more reliable results enabled launching a rapid cholera alert and response in Kasese district. Besides being suitable for use in remote areas, the enriched test had several additional benefits.

First, the early cholera alert facilitated preparations for managing the outbreak within the district which led to relatively fewer cholera causes and shorter duration of the epidemic (16 weeks) compared to previous years (where outbreak lasted for over 9 months) [25].

Second, the dipstick test allowed the hospitals and the district staff to monitor the patients and the course of the outbreak more closely than ever before. There was quick action since laboratory results were received the same day. Unlike in the previous outbreaks where the normal practice was to transport random stool samples to Kampala or laboratory within the region and wait for 3–7 days to get laboratory report to guided the next steps [22], the test were done within the health facility and results received within a day.

Third, there was better use of laboratory reports such as recognizing that even during cholera outbreaks; some cases with diarrhea who met the standard case definition may be due to other causes and required immediate referral to other sections within the hospital.

Even though reliable test results were quicker to get and available the same day compared to culture method that took more days, health workers in Kasese district still asked for a shorter time period similar to that for malaria RDTs in which the time was 15–20 min [26, 27].

The authors recommend that in order to strengthen the management of cholera in similar manner to other infectious diseases of public health importance such as malaria, a rapid and reliable diagnostic test should be an essential component of cholera prevention and control efforts.

Therefore, the use of the dipstick will not totally replace culture since culture is still needed to confirm epidemics, to monitor antibiotic sensitivity and to produce pure isolates for molecular characterization. However, the enriched dipstick method can be a valuable tool for detecting and monitoring cholera outbreaks.

Furthermore, epidemiological description of the outbreak using more reliable test highlighted important observations. First, although all ages were affected, we also noted that the younger age groups, up 19 years were the majority in the patient registers. Since this age group includes the school age, we think that cholera could contribute significantly to school absenteeism since studies on cholera in Kasese district showed that cholera was an endemic disease occurring frequently in some sub-counties within the district [28].

Second, the high proportion of women in the reproductive years was an indication of increased risk for this age, sex group. Therefore, provision of targeted health education and the use of Oral Cholera Vaccines (OCV) for these groups and other eligible ones in endemic districts such as Kasese [28] could be explored as recommended by the current WHO guidelines [29]. Since global demand for OCV outweigh the needs [30] targeting the most at risk/vulnerable groups namely; the children under 9 years and the women should result in maximum benefit from integrated cholera prevention and control interventions.

Third, though the case fatality rate was low among the patients in this study, the death that occurred while a patient was being treated on the medical ward emphasizes the need for careful observation and documentation of fluid losses for patients with severe watery diarrhea. The earlier use of the rapid test might have alerted the health personnel that the patient had cholera; however, it should be noted that cholera is not the only cause of severe diarrhea and rehydration treatment depends on the symptoms and not on the result of the rapid test.

Forth, it was worth noting that most of the cases detected in this study came from sub-counties close to the health facilities as shown in Fig. 3. Since cholera outbreaks are rarely so focused near health facilities, it seems likely that these outbreaks also affected communities further away but may not have come to the designated cholera treatment centres or were treated but not reported.

The authors therefore recommend follow-up studies to determine the following: the source of the cholera outbreak; the explanation for the observed age and sex distribution to why more males below the age of 20 years were affected than the female counterparts and why more adult females were affected and a study on actual impact of cholera during an outbreaks such as the one describe in this study since the cases being treated at the health facility may represent a portion of the actual number of cases and deaths.

Finally, while results after 6 h and within a day was useful for decision making in remote areas, there is a need for rapid and a reliable test that provides results more quickly, similar to the malaria test which gives test results within 20 min [26, 27].

## Conclusions

Our findings showed that the modified RDT test after enrichment with 1% alkaline peptone water had high level of accuracy in detection of *V. cholerae* and is quick, affordable alternative cholera outbreak monitoring tool that should be put to wider use in resource constrained settings. However, culture method should remain for cholera outbreak/epidemic confirmation, to monitor antibiotic sensitivity and to produce pure isolates for molecular characterization.

## Abbreviations

AKD: Amanda Kay Debes
APW: Alkaline Peptone Water
AR: Attack rate
CFR: Case fatality rate
CGO: Christopher Garimoi Orach
CPHL: Central Public Health Laboratory
DAS: David A. Sack
DRC: Democratic Republic of Congo
GB: Godfrey Bwire
HIV: Human immunodeficiency virus
IRB: Institution Review Board
MLTC: Mpondwe-Lubirikha Town Council
MR: Malathi Ram
RDT: Rapid diagnostic test
TC: Town Council
UBOS: Uganda Bureau of Statistics
WHO: World Health Organization
